# Transcriptional Profiling Reveals the Regulatory Role of *CXCL8* in Promoting Colorectal Cancer

**DOI:** 10.3389/fgene.2019.01360

**Published:** 2020-01-21

**Authors:** Jie Li, Qin Liu, Xuan Huang, Yurui Cai, Li Song, Qianrong Xie, Fuchuan Liu, Xiaochun Chen, Peng Xu, Fanwei Zeng, Yanpeng Chu, Fanxin Zeng

**Affiliations:** ^1^ Department of Clinical Research Center, Dazhou Central Hospital, Dazhou, China; ^2^ Medical Research Center, Beijing Chaoyang Hospital, Capital Medical University, Beijing, China; ^3^ Department of Pathology, Dazhou Central Hospital, Dazhou, China; ^4^ School of Medicine, Sichuan University of Arts and Science, Dazhou, China

**Keywords:** colorectal cancer, RNA-sequencing, *CXCL8*, cell proliferation and differentiation, apoptosis, cytokines

## Abstract

C-X-C motif chemokine ligand 8 (*CXCL8*) is involved in tumor proliferation, migration, and invasion. However, the function of *CXCL8* in colorectal cancer (CRC) is controversial. Here, we analyzed RNA-sequencing (RNA-seq) data to identify differentially expressed genes and pathways according to gene ontology (GO) enrichment and Kyoto Encyclopedia of Genes and Genomes (KEGG) pathways associated with CRC. The levels of the mRNA encoding *CXCL8* were significantly increased in early and advanced stages of CRC, as well as in metastases and nonmetastasis cases using RNA-seq analysis (n = 91). These findings were consistent with immunohistochemical analysis of *CXCL8* expression (n = 87). Protein-protein interaction (PPI) prediction combined with transcriptional profiling data revealed that *CXCL8* levels positively correlated with cAMP responsive element binding protein 1 (*CREB1*)/ribosomal protein S6 kinase B1 (*RPS6KB1*) expression, which promotes cell proliferation and differentiation in high expression, while inversely correlated with the expression of Bcl2 associated agonist of cell death (*BAD*) protein to inhibit apoptosis during the progression of CRC. These findings provide compelling clinical and molecular evidence to support the conclusion that *CXCL8* contributes to the genesis and progression of CRC.

## Introduction

Colorectal cancer (CRC) ranks third and second worldwide among cancers of males and females, respectively (Torre et al., 2015). Every year, about 1 million people worldwide suffer from CRC, and more than 490,000 people die from CRC (Weitz et al., 2005). In China, the annual number of new CRC may exceed 300,000, and the incidence is gradually increasing (Chen et al., 2015). Generally, the prognosis of patients with early-stage CRC (stages I and II) is better compared with those with advanced CRC (stages III and IV). For example, the 5-year survival rates of patients with stage I CRC is approximately about 90% in striking contrast to ≤12% for patients with stage IV CRC (Miller et al., 2019), which indicates the critical importance of identifying in greater detail the mechanisms underlying the cause of CRC and its progression.

Chemokine families comprise structure related signaling molecules that mediate physiological process through recruiting and activating that guide the movement of leukocytes associated with inflammation (Luster, 1998; Locati and Murphy, 1999). Studies have shown that chemokines can regulate the proliferation of tumor cells and mediate the infiltration of tumors with immune cells (Tanaka et al., 2005; Bindea et al., 2014; Ma et al., 2014). The C-X-C motif chemokine ligand 8 (*CXCL8*), which is a member of the CXC chemokine family, is a pro-inflammatory cytokine produced by neutrophils, macrophages, endothelial cells, and cancer cells (Kitadai et al., 1998; Inoue et al., 2000; Konno et al., 2003 ). The levels of *CXCL8* are elevated in gastric, breast, and pancreatic cancers (Konno et al., 2003; Bellone et al., 2006; Derin et al., 2007). Furthermore, elevated *CXCL8* expression is associated with the induction of angiogenesis as well as increased proliferation, invasion and migration of tumor cells (Singh et al., 1999; Inoue et al., 2000; Zhu et al., 2004; Matsuo et al., 2009; Fernando et al., 2011; Ning et al., 2011; Roshani et al., 2014; Liu et al., 2016). Ning Y. et al. found that *CXCL8* promotes the proliferation and metastasis of a CRC cell line (Ning et al., 2011). Although the physiological and pathological function of *CXCL8* have been the subject of intensive investigations for decades, its functions in the pathogenesis and progression of CRC is controversial.

We therefore aimed to identify the role of *CXCL8* using transcription profiling and through the analysis of PPI networks of large clinical cohort.

## Materials and Methods

### Patients and Tissue Samples

Three independent cohorts of 187 patients diagnosed with CRC were included in the study as follows: Cohort 1, including nine patients (six males and three females), with a median age (years) of 65 ranging from 45 to 80; and Cohort 2, including 91 patients (54 males and 37 females), with a median age 61 ranging from 30 to 85. Patients underwent surgery at Dazhou Central Hospital from January 2018 to March 2019. Diagnoses of CRC were histopathologically confirmed. Patients diagnosed with CRC underwent radical resection of the primary tumor. The clinical stage of the tumor was determined according to the tumor-node-metastasis (TNM) staging system. CRC tissues and their matched normal tissues were collected for RNA-seq analysis and were stored in liquid nitrogen immediately after surgery. Cohort 3, including 87 patients (59 males and 28 females) with a median age of 63 ranging from 42 to 83, underwent treatment similar to that of Cohorts 1 and 2. Their tumor and adjacent normal tissues were acquired as paraffin-embedded samples from the Department of Pathology. These tissues were harvested from patients treated from 2014 to 2018. The three cohorts had no difference in age (Kruskal-Wallis test, *P* = 0.364) and sex (Chi-square test, *P* = 0.492) distribution. The patients in metastasis group are those who occurred new metastasis during at least 3 months of follow-up. The new metastasis only included distant metastasis. Patients were required to grant their written informed consent to be included in the study. The Medical Ethics Review Board of Dazhou Central Hospital approved the study (IRB00000003-17003).

### RNA-Seq and Data Analysis

Total RNA was extracted using TRIZOL reagent (Takara Biomedical Technology, Beijing, China). An Agilent 2100 RNA Nano 6000 Assay Kit (Agilent Technologies, CA, USA) was used to detect the integrity and concentration of total RNA. After the total RNA samples were qualified, magnetic beads with oligo (dT) were selected for enrichment and purification. The synthesized chains were purified by QIAquick PCR purification kit and eluted by EB buffer. After that, the purified double-stranded cDNAs was subjected to end-repairing, base A, and sequencing ligation. Then the target fragment was recovered by agarose gel electrophoresis and amplified by PCR. Qualified libraries were sequenced using Illumina platform with sequencing strategy PE150. Raw reads obtained from Illumina platform sequencing were processed by removing low-quality sequences and joint contamination to get high-quality sequences (clean reads). The subsequent analyses were based on clean reads.

### GO Enrichment, KEGG Pathway, and PPI Analyses

The functional roles of differentially expressed genes (DEGs) were revealed by determining the transcriptional profiles acquired using RNA-seq and by GO and KEGG enrichment analyses. We used the GO database to analyze the functional enrichment of DEGs, focusing on the pathways associated with these genes that were enriched in the terms biological process, molecular function and cellular component. The KEGG pathway database was used to determine the enrichment of DEGs. The threshold of the hypergeometric distribution test for default enrichment results was 0.05. The PPI network of DEGs was constructed according to information acquired using the STRING database (https://string-db.org/). To identify hub genes in the PPI network, we implemented maximal clique centrality analysis into *cytoHubba* (a Cytoscape plugin). Maximal clique centrality is a topological analytical method that effectively screens for hub genes (Chin et al., 2014).

### Immunohistochemistry (IHC) and Pathology Scores

Paraffin-embedded tumor and adjacent normal tissues of patients with CRC were cut into 4-μm-thick sections. Antibodies against *CXCL8* (1:100, Affinity, USA) were used to analyze the sections. IHC staining was performed according to the manufacturer’s instructions. The percentages of positive cells were classified as follows (percentage scores): < 5% (0), 5%–25% (1), 25–50% (2), 50–75% (3), and > 75% (4). The staining intensities were classified as follows (intensity scores): negative (0), weak (1), moderate (2), and strong (3). The final score was determined by the following formula: total score = percentage scores × intensity scores. Total scores ranging from 0 to 4 were defined as low group, and total scores ranging from 5 to12 were defined as the high group.

### Statistical Analysis

DEGs were analyzed using the DESeq2 package in R Studio (Version: 3.6.1). |log2 Fold change|≥1, and *p*-adjust value < 0.05 was chosen as the cut-off value for identifying DEGs. The Mann-Whitney U test was performed to evaluate the significance of differences between two groups. All statistical analyses were performed using SPSS 20.0. The data were presented as the mean ± SEM, and *p* < 0.05 indicates a significant difference.

## Results

### DEGs Associated With CRC

**Figure1:**
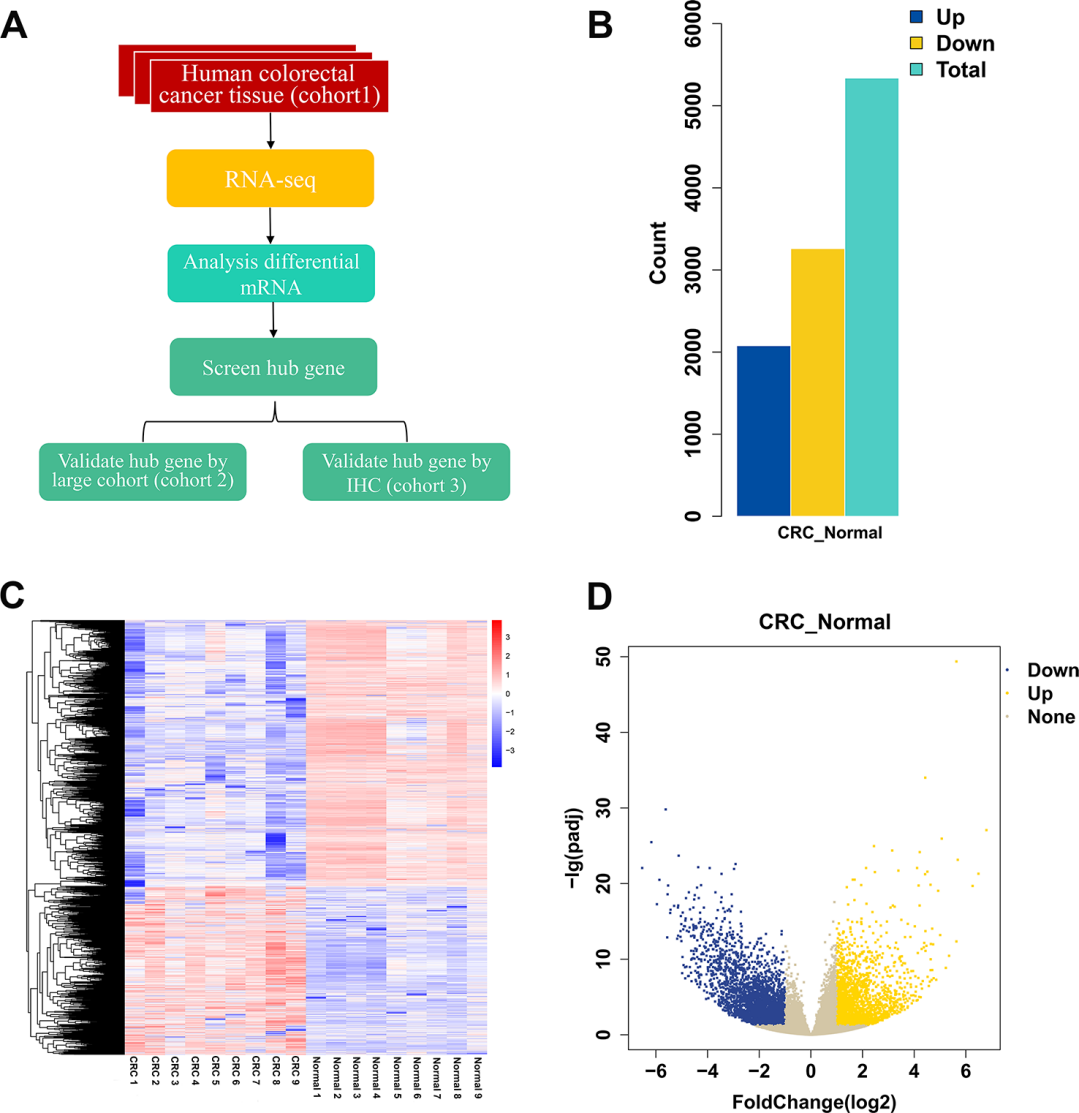
Differential gene expression between cancer and normal tissues of patients with CRC. **(A)** Workflow of the study. **(B)** The numbers of upregulated and downregulated genes. **(C)** Heatmap of differentially expressed genes between normal group and CRC group. **(D)** Volcano plot of differentially expressed mRNAs between normal group and CRC group. N = 9. CRC, colorectal cancer.

The workflow of the study is shown in **Figure 1A**. To identify DEGs, we performed RNA-seq in nine patients with CRC. We identified 5,338 DEGs of which 2,078 were upregulated genes and 3,260 were downregulated genes (**Figure 1B**). Top 20 DEGs between normal tissues and tumor tissues of patients with CRC are present in **Table 1**. Heatmaps and volcano plots of the distributions of DEGs are displayed in **Figures 1C, D**. Compared with the levels of expression of the matched normal tissues, the levels of *CXCL8* mRNA were found to be one of the most upregulated genes among tumor tissues (log2 Fold change = 3.45, *p*-adjust value = 2.71×10^-8^).

**Table 1:** Top 20 differentially expressed genes between normal tissues and tumor tissues of patients with colorectal cancer (CRC).

ENSG	Gene name	Log2Fold change	*P*-adj	UP/Down	ENSG	Gene name	Log2Fold change	*P*-adj	UP/Down
ENSG00000170373	CST1	6.796632	8.55E-28	Up	ENSG00000167676	PLIN4	−5.62135	1.54E-30	Down
ENSG00000115593	SMYD1	−6.53111	8.70E-23	Down	ENSG00000176194	CIDEA	−5.56264	1.31E-13	Down
ENSG00000134827	TCN1	6.488626	4.93E-22	Up	ENSG00000184811	TUSC5	−5.54646	2.62E-19	Down
ENSG00000123500	COL10A1	6.261218	2.06E-20	Up	ENSG00000104833	TUBB4A	−5.52692	1.79E-20	Down
ENSG00000135447	PPP1R1A	−6.17527	3.32E-26	Down	ENSG00000171246	NPTX1	−5.44691	7.69E-17	Down
ENSG00000089250	NOS1	−5.96224	5.35E-18	Down	ENSG00000130226	DPP6	−5.43224	2.88E-17	Down
ENSG00000224958	PGM5-AS1	−5.86345	3.17E-21	Down	ENSG00000108244	KRT23	5.350705	3.53E-11	Up
ENSG00000015413	DPEP1	5.686867	7.12E-24	Up	ENSG00000034971	MYOC	−5.29445	8.80E-18	Down
ENSG00000228742	RP5-884M6.1	5.634692	4.51E-13	Up	ENSG00000251026	RP11-138J23.1	5.224942	1.40E-09	Up
ENSG00000164283	ESM1	5.63467	4.26E-50	Up	ENSG00000181195	PENK	−5.17554	1.94E-13	Down

### GO and KEGG Analyses of DEGs

**Figure 2:**
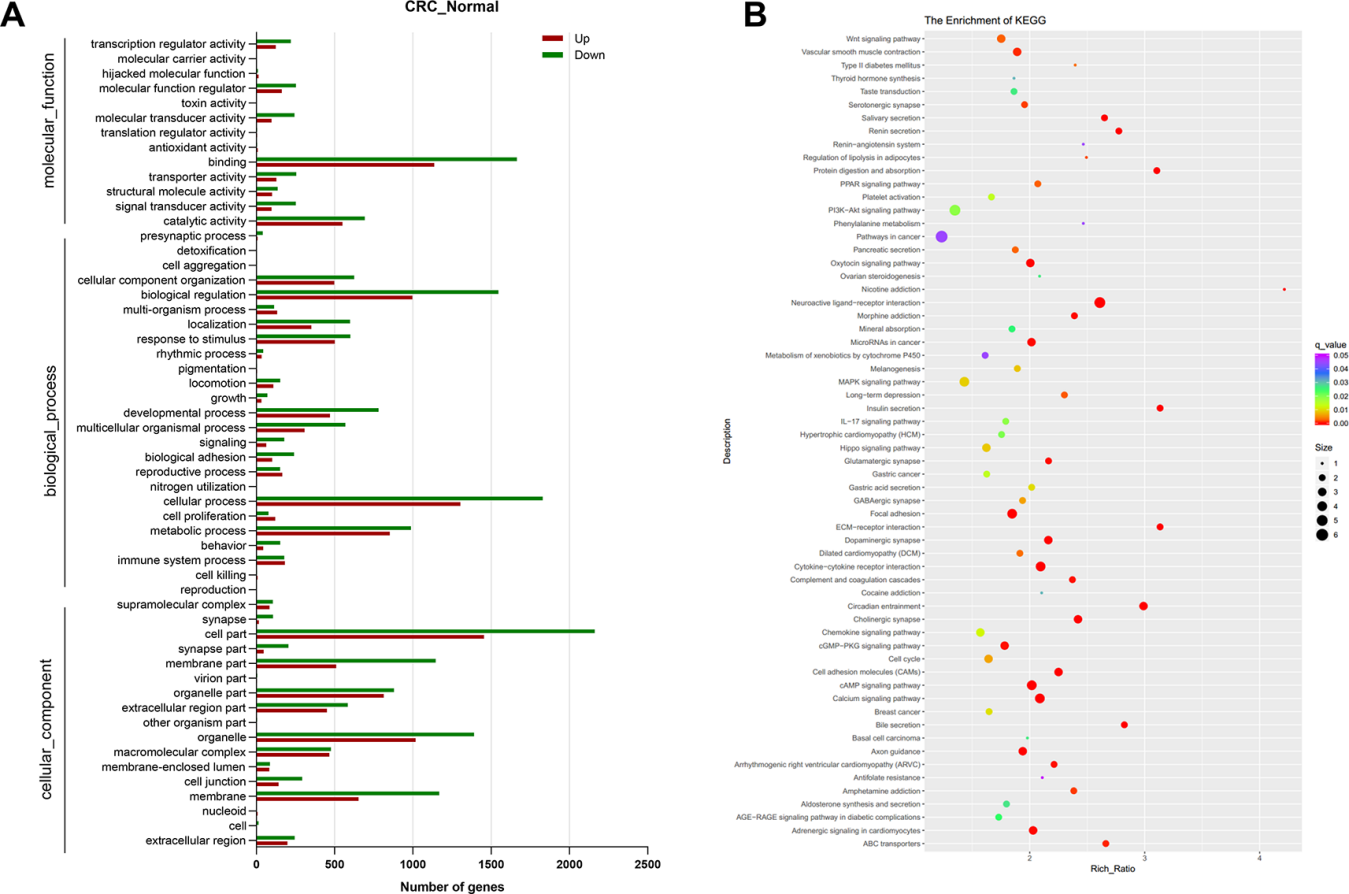
Functional analysis of the differentially expressed genes. **(A)** Gene ontology (GO) enrichment and **(B)** Kyoto Encyclopedia of Genes and Genomes (KEGG) pathway enrichment analysis of differentially expressed genes between normal group and colorectal cancer (CRC) group. N = 9.

To further illustrate the function of DEGs in human CRC, GO, and KEGG pathway enrichment analysis was performed. GO analysis identified 13,763 GO terms enriched for the DEGs, of which 1,697 were significantly enriched, including terms related to molecular function, biological process and cellular component. The top 55 significantly enriched GO terms in level 2 are shown in **Figure 2A**. Molecular function analysis showed that the top two terms of DEGs were related to binding (GO:0005488) and catalytic activity (GO:0003824). In the category of biological process, most DEGs were associated with cellular process (GO: 0009987). There were 62 pathways with the q-value < 0.05 figured out by KEGG pathway analysis of DEGs showed in **Figure 2B**. Among them, the six most significantly enriched pathways were Neuroactive ligand-receptor interaction, Circadian entrainment, ECM-receptor interaction, Insulin secretion, Protein digestion and absorption, and Cytokine-cytokine receptor interaction, for which there were 93, 43, 37, 37, 37, and 67 enriched DEGs, respectively. In Cytokine-cytokine receptor interaction pathways, *CXCL8* was illustrated to be highly relevant to cancer.

### High Expression Levels of *CXCL8* in CRC

**Figure 3:**
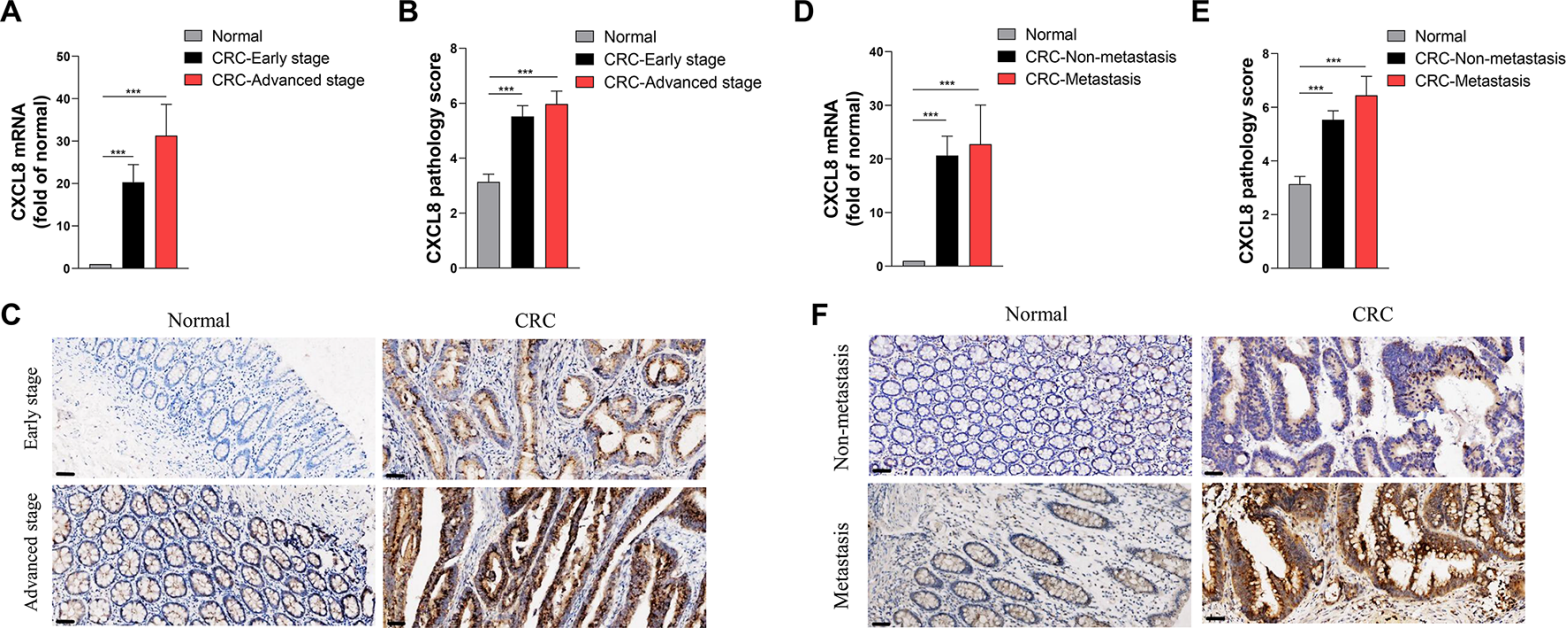
Expression levels of *CXCL8* in the clinical cohort. **(A)** The levels of *CXCL8* mRNA in the normal (N = 81), colorectal cancer (CRC)-early stage (N = 44) and CRC-advanced stage (N = 37) groups. **(B)** The levels of *CXCL8* protein in normal (N = 87), CRC-early stage (N = 48), and CRC-advanced stage (N = 39) groups. **(C)** Immunohistochemical staining of normal, CRC-early stage and CRC-advanced stage groups. **(D)** The levels of *CXCL8* mRNA in normal (N = 70), CRC-nonmetastasis (N = 58) and CRC-metastasis (N = 12) groups. **(E)** The levels of *CXCL8* protein in normal (N = 87), CRC-nonmetastasis (N = 69) and CRC-metastasis (N = 18) groups. **(F)** Immunohistochemical staining of normal, CRC-nonmetastasis and CRC-metastasis groups. Scale bar = 50 μm. Statistical signiﬁcance was performed by the Mann-Whitney U test. ***p < 0.001.

In order to rank and score nodes by network features, hub genes were assessed by maximal clique centrality analysis in *cytoHubba*, which identified *CXCL8* as a candidate (**Supplementary Figure 1**). Next, we validated the *CXCL8* expression levels in Cohort 2. The RNA-seq profiles revealed that the levels of *CXCL8* mRNA in tumor, compared with those of normal tissues, were significantly increased in patients with early-stage or advanced-stage in CRC (**Figure 3A**). IHC analysis of CRC and matched normal tissues of Cohort 3 revealed that the levels of *CXCL8* were notably increased in tumors versus normal tissues, and the expressions of *CXCL8* in advanced-stage CRC were insignificant different compared with those of early-stage CRC (**Figures 3B, C**).

Similarly, we used RNA-seq and IHC to determine the levels of *CXCL8* mRNA and protein respectively, in patients with nonmetastatic versus metastatic CRC. The levels of *CXCL8* mRNA were significantly higher in CRC tissues compared with those of normal tissues, and the metastasis group expressed higher levels of *CXCL8* mRNA compared with the nonmetastasis group, although the differences were not significant (**Figures 3D–F**). The RNA-seq data were corroborated by IHC results. Furthermore, there was no significant difference between *CXCL8* protein levels in tumor size < 5 cm and ≥5 cm groups, as well as those of mass and ulcer types of CRC groups (**Supplementary Figures 2A, B**). Notably, the proportions of abnormal carcinoembryonic antigen (CEA) and carbohydrate antigen 19-9 (CA19-9) were increased in line with *CXCL8* expression level through IHC assays (**Supplementary Figures 3A, B**). These results suggested that *CXCL8* plays an important role in CRC progression.

### *CXCL8* Levels Correlate With Those of *CREB1*, *RPS6KB*, and *BAD* During CRC Progression

**Figure 4:**
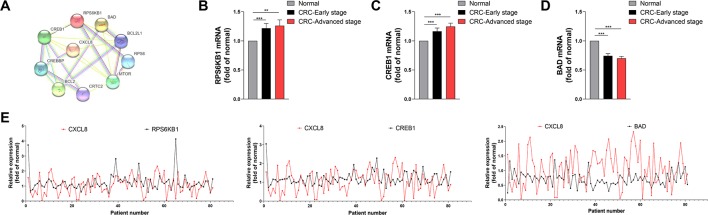
Expression of *RPS6KB1* and *CREB1* is upregulated and positively correlates with *CXCL8* expression, while Bcl2 associated agonist of cell death (*BAD)* is downregulated and negatively correlates with *CXCL8* expression in colorectal cancer (CRC). **(A)** Protein-protein interaction (PPI) network. **(B)** Expression of *RPS6KB1* mRNA expression in normal (N = 81), CRC-early stage (N = 44), and CRC-advanced stage (N = 37) groups. **(C)** Expression of *CREB1* mRNA expression in normal (N = 81), CRC-early stage (N = 44) and CRC-advanced stage (N = 37) groups. **(D)** Expression of *BAD* mRNA expression in Normal (N = 81), CRC-early stage (N = 44), and CRC-advanced stage (N = 37) groups. **(E)** Expression of *RPS6KB1*, *CREB1*, *BAD* mRNAs compared with that of *CXCL8* mRNA in CRC (N = 81). Statistical signiﬁcance was performed by the Mann-Whitney U test. **p < 0.01, ***p < 0.001.

In order to analysis the protein interaction network, PPI was constructed by STRING online. Network analysis revealed that the *CXCL8* network was mainly involved in regulating signaling pathways required for cell proliferation, differentiation and apoptosis (**Figure 4A**). RNA-seq analysis indicated that the levels of the mRNAs encoding *RPS6KB1* and *CREB1* were significantly increased in CRC tissues compared with those of normal tissues during early and advanced stages of CRC (**Figures 4B, C**). In contrast, *BAD* expression was significantly decreased in CRC tissues during these stages (**Figure 4D**). Moreover, the levels *CREB1* and *RPS6KB1* mRNAs positively correlated with those of *CXCL8* mRNA, while the levels of *BAD* mRNA negatively correlated with those of *CXCL8* mRNA (**Figure 4E**). The mRNA levels of *RPS6KB1* and *CREB1* were significantly increased in CRC-nonmetastasis compared with those in normal group. But no significance was observed between CRC-metastasis group and normal group. *BAD* showed significant difference between CRC-nonmetastasis and normal group, as well as CRC-Metastasis and normal group (**Supplementary Figure 4**). Taken together, these findings indicate that *CXCL8* may upregulate the expression of *CREB1* and *RPS6KB1* to promote cell proliferation and differentiation, while downregulating *BAD* expression to inhibit apoptosis during the progression of CRC.

## Discussion

*CXCL8* is very important chemokine in determining inflammation or immune response (Ha et al., 2017). However, its roles in CRC remain largely unknown. In this study, we identified 2,078 upregulated and 3,260 downregulated DEGs in patients with CRC. GO and KEGG enrichment analysis indicated that cytokine-cytokine receptor interaction pathway played an essential role in CRC progression and that *CXCL8* represented a core hub gene in the network. We further validated the upregulation of *CXCL8* in both mRNA and protein levels in CRC tissues, based on our large clinical cohorts that involved total 187 cases through RNA-seq data combined with IHC analysis. Notably, the levels of *CREB1*, *RPS6KB1,* and *BAD* mRNAs correlated well with those of *CXCL8* expression, suggesting that *CXCL8* is essential for mediating the progression of CRC.

High serum levels of *CXCL8*, which serve as a protective barrier for liver metastasis of CRC, are associated with better prognosis (Fichtl et al., 2016; Wang et al., 2017). On the contrary, studies have shown that elevated levels of *CXCL8* could promote the carcinogenesis associated with poor prognosis (Cheng et al., 2014). Therefore, the role of *CXCL8* in CRC remains controversial. In our study, high levels of *CXCL8* in differentially expressed were observed in early- and advanced-stage CRC as well as in tissues of patients with nonmetastatic and metastatic tumors. However, there was no significant difference between the levels of *CXCL8* expressed by these subgroups. Other experiments demonstrated that *CXCL8* has no significant correlation between protein levels and tumor size (Xiao et al., 2015). In our cohort, both the tumor size and the tumor type did not affect the expression of *CXCL8*. Notably, high *CXCL8* expression was associated with higher abnormal proportion of CEA and CA19-9 in CRC.

A large body of evidence shows that the increase cell proliferation and differentiation and decrease of cell apoptosis play an important role in the genesis and progression of cancers (Thompson, 1995). In the present study, PPI analysis shows that *RPS6KB1* and *CREB1*, which are associated with proliferation and differentiation, as well as the proapoptotic protein *BAD*, may interact with *CXCL8*. In addition, the levels of *RPS6KB1* and *CREB1* mRNAs were increased in tissues harvested from patients with the early and advanced stages of CRC, while those of *BAD* was decreased. *RPS6KB1* and *CREB1* expression were positively correlated with that of *CXCL8*, whereas there was an inverse correlation between *BAD* and *CXCL8* expression. Our findings were consistent with the existed evidence indicating that the genes that mediate cell proliferation and differentiation were upregulated while proapoptotic genes were downregulated during tumor progression (Xiao et al., 2015; Fisher et al., 2019). These results suggest that *CXCL8* may regulate the expression of *RPS6KB1* and *CREB1* to promote cell proliferation and differentiation while inhibiting *BAD* expression to suppress apoptosis of CRC cells.

Additionally, there are also some limitations need to be acknowledged in this study. Firstly, these findings are based on a single-center study. More samples from multicenter are needed to further support our findings. Secondly, animal or cell experiment would be required to further confirm the mechanism of gene expression and phenotype.

## Conclusion

Our study demonstrates that *CXCL8* plays an important role in the progression of CRC by mediating proliferation, differentiation, and apoptosis within a regulatory network. These findings indicate that *CXCL8* may serve as a target for therapy of CRC.

## Data Availability Statement

This article contains unpublished data. The name of the repository is NCBI SRA database and the accession number is PRJNA591037.

## Ethics Statement

The studies involving human participants were reviewed and approved by The medical ethics review board of Dazhou Central Hospital. The patients/participants provided their written informed consent to participate in this study.

## Author Contributions

FXZ, YPC, and JL participated in the study design and manuscript preparation. JL, QL, and XH performed RNA-seq data analysis and prepared figures. LS, QX, and FWZ collected the detailed information of patients. YRC and QL performed immunohistochemistry staining and analyzed the related data. FL, XC, and PX prepared and cut paraffin-embedded tissues into slices. All authors read and approved the final draft.

## Funding

This work was supported by the National Natural Science Foundation of China (81902861), the Scientific Research Fund of Sichuan health and Health Committee (No. 18PJ361), and the Scientific Research Fund of Science and Technology Bureau in Sichuan Province (No. 2018138, No. 2018JY0324).

## Conflict of Interest

The authors declare that the research was conducted in the absence of any commercial or financial relationships that could be construed as a potential conflict of interest.

## Supplementary Material

Supplementary Figure 1 Top 10 hub genes calculated by maximal clique centrality in Cytoscape.

Supplementary Figure 2
**(A)** The relation between *CXCL8* protein expression and tumor size. Tumor size < 5 cm (N = 64), tumor size≥5 cm (N = 23). **(B)** The relation between *CXCL8* protein expression and tumor type. Mass type (N = 45), ulcer type (N = 41).

Supplementary Figure 3
**(A)** The relation between *CXCL8* protein expression and CEA. **(B)** The relation between *CXCL8* protein expression and CA19-9. CEA, carcinoembryonic antigen; CA19-9, carbohydrate antigen 19-9.

Supplementary Figure 4
**(A)** Expression of *RPS6KB1* mRNA expression in normal (N = 70), CRC-nonmetastasis (N = 58) and CRC-metastasis (N = 12) groups. **(B)** Expression of *CREB1* mRNA expression in normal (N = 70), CRC-nonmetastasis (N = 58) and CRC-metastasis (N = 12) groups. **(C)** Expression of *BAD* mRNA expression in Normal (N = 70), CRC-nonmetastasis (N = 58) and CRC-metastasis (N = 12) groups. Statistical signiﬁcance was performed by the Mann-Whitney U test. ****p* < 0.001.
