# Supplementary figures and images for: Transcriptional Profiling Reveals the Regulatory Role of CXCL8 in Promoting Colorectal Cancer
Source: Front Genet. 2020 Jan 21;10:1360. doi: 10.3389/fgene.2019.01360 (PMC6985586; doi:10.3389/fgene.2019.01360)

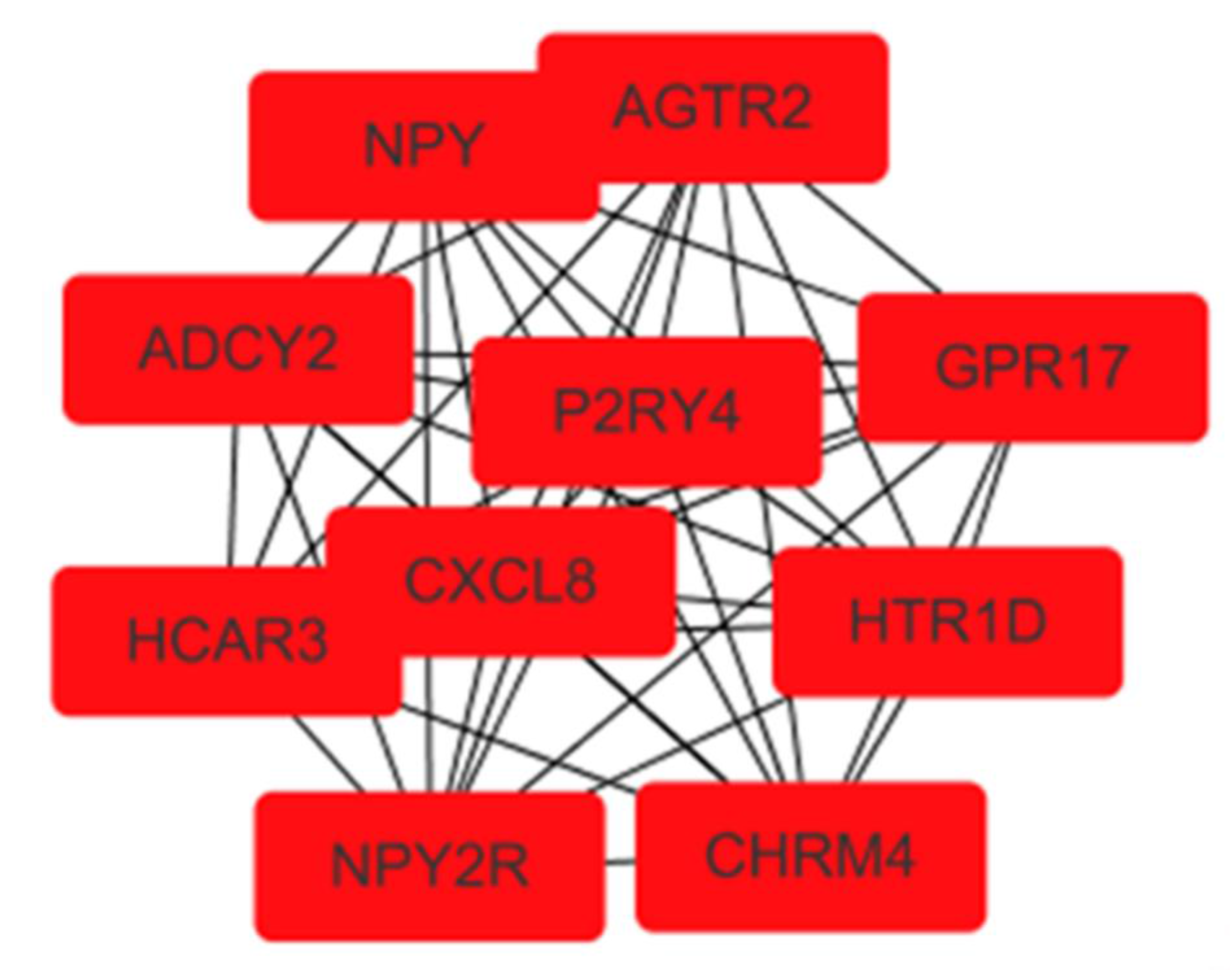

Supplement: Supplementary Figure 1 — Top 10 hub genes calculated by maximal clique centrality in Cytoscape. [file Image_1.tif]

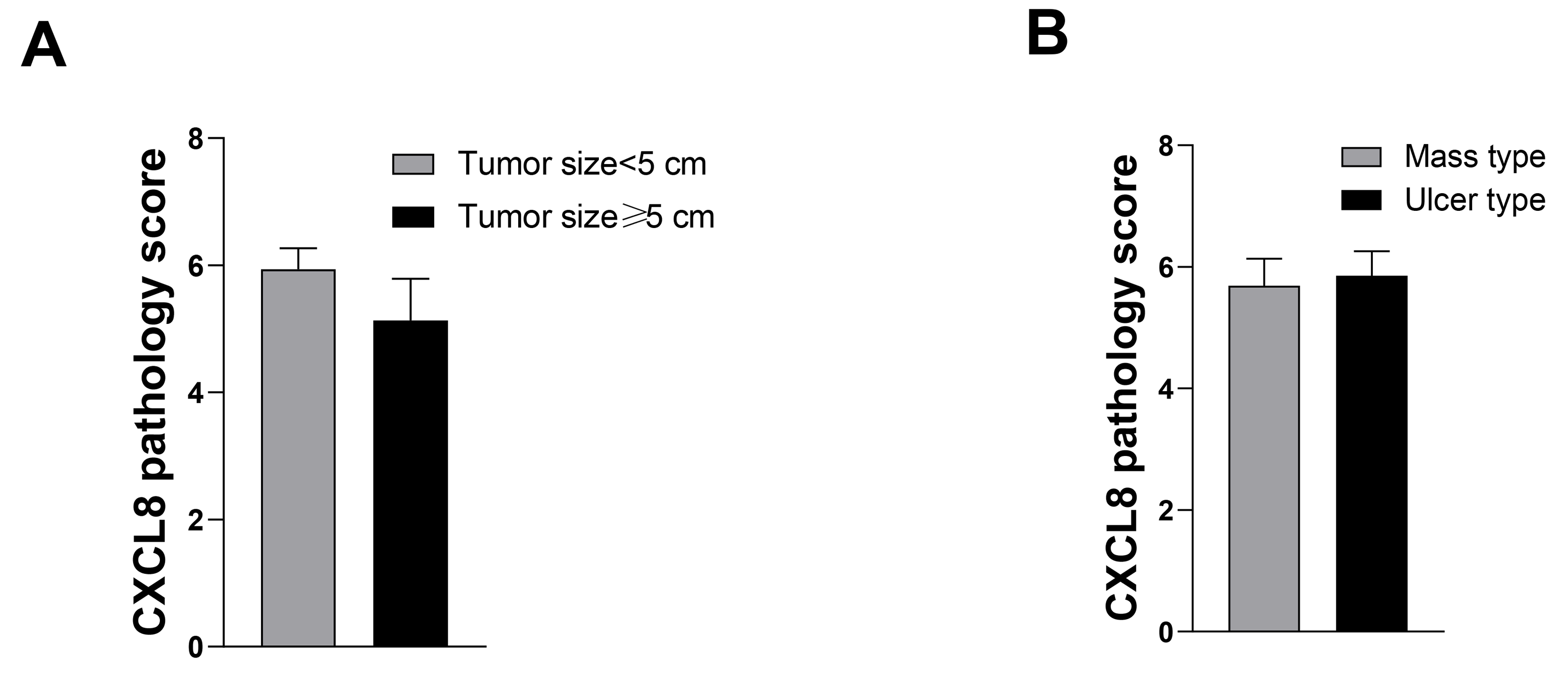

Supplement: Supplementary Figure 2 — (A) The relation between CXCL8 protein expression and tumor size. Tumor size < 5 cm (N = 64), tumor size≥5 cm (N = 23). (B) The relation between CXCL8 protein expression and tumor type. Mass type (N = 45), ulcer type (N = 41). [file Image_2.tif]

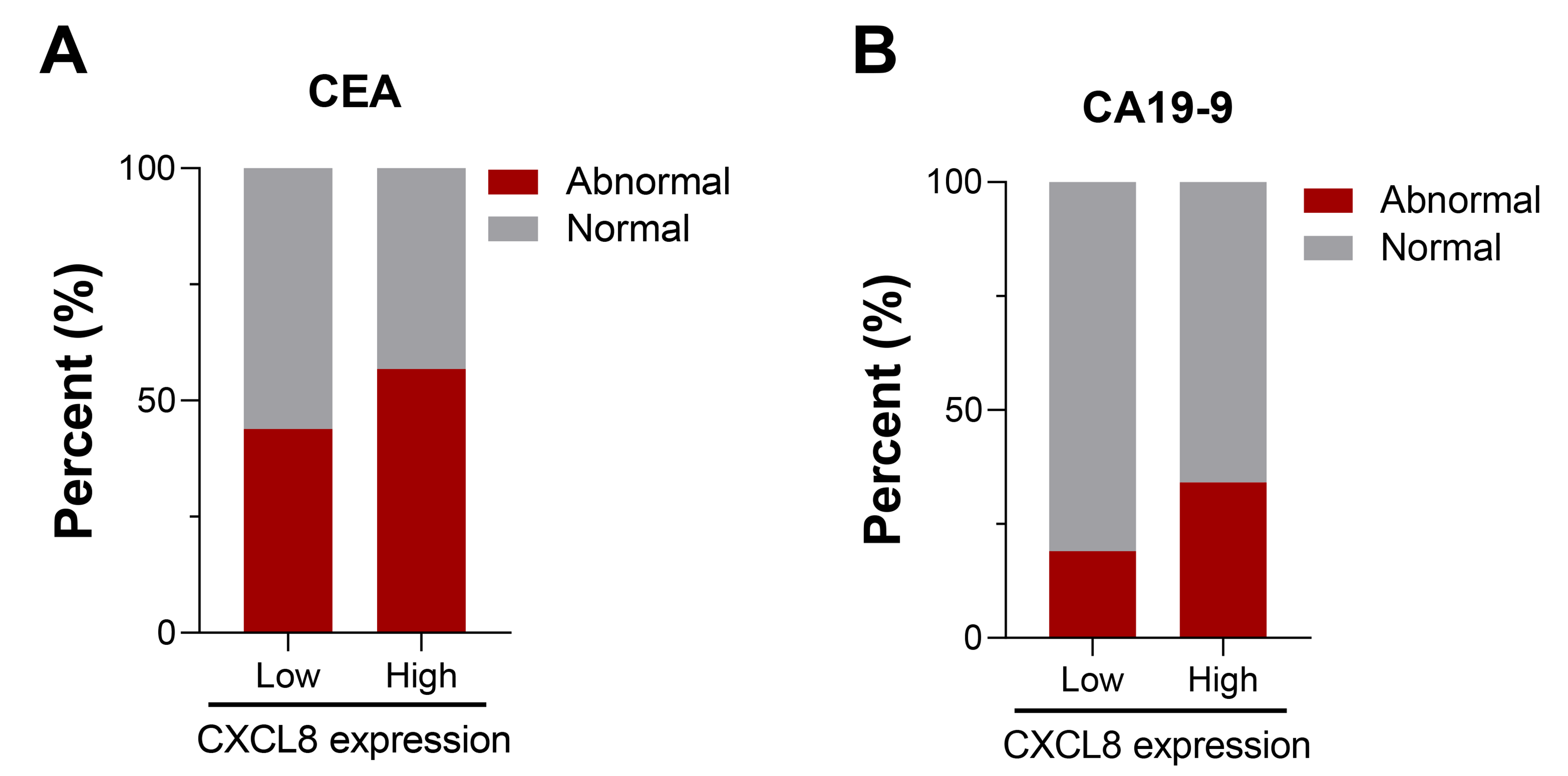

Supplement: Supplementary Figure 3 — (A) The relation between CXCL8 protein expression and CEA. (B) The relation between CXCL8 protein expression and CA19-9. CEA, carcinoembryonic antigen; CA19-9, carbohydrate antigen 19-9. [file Image_3.tif]

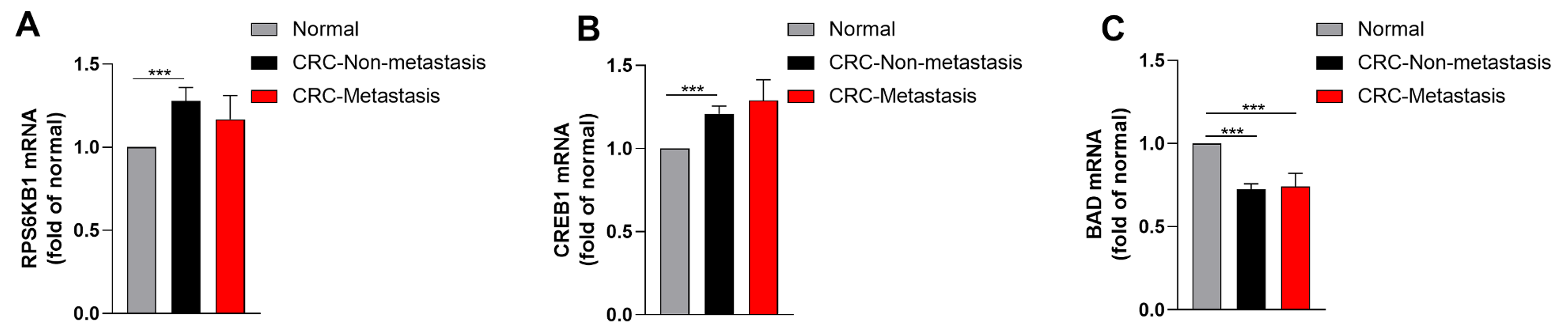

Supplement: Supplementary Figure 4 — (A) Expression of RPS6KB1 mRNA expression in normal (N = 70), CRC-nonmetastasis (N = 58) and CRC-metastasis (N = 12) groups. (B) Expression of CREB1 mRNA expression in normal (N = 70), CRC-nonmetastasis (N = 58) and CRC-metastasis (N = 12) groups. (C) Expression of BAD mRNA expression in Normal (N = 70), CRC-nonmetastasis (N = 58) and CRC-metastasis (N = 12) groups. Statistical signiﬁcance was performed by the Mann-Whitney U test. ***p < 0.001. [file Image_4.tif]
